# Changes of AgNORs in HeLa cells during serum starvation.

**DOI:** 10.1038/bjc.1990.303

**Published:** 1990-09

**Authors:** K. Matsumura, K. Sasaki, T. Tsuji, T. Murakami, M. Takahashi, F. Shinozaki

**Affiliations:** Department of Oral & Maxillofacial Surgery, Yamaguchi University School of Medicine, Ube, Japan.

## Abstract

**Images:**


Br. J. Cancer (1990), 62, 385-387 © Macmillan Press Ltd., 1990

### Changes of AgNORs in HeLa cells during serum starvation

K. Matsumura[1], K. Sasaki[2], T. Tsuji[1], T. Murakami[2], M. Takahashi[2] & F. Shinozaki[1]

*Departments of [1]Oral & Maxillofacial Surgery and [2]Pathology Yamaguchi University School of Medicine, Ube 755, Japan.*

Nucleolar organiser regions (NORs) have recently attracted much attention because of evidence that the number of silver-stained NOR sites (AgNORs) within nuclei is significantly higher in malignant cells than in normal, reactive, or benign neoplastic cells (Ploton *et al.*, 1986; Howat *et al.*, 1988; Smith & Crocker, 1988; Matsumura *et al.*, 1989; Suarez *et al.*, 1989). Furthermore, the number of AgNORs appears to correlate with various parameters of cellular activity. The mean number of AgNORs was shown to be linearly related to the proportion of tumour cells with nuclear Ki-67 immunoreactivity (Hall *et al.*, 1988) or with the size of the S-phase fraction estimated by DNA flow cytometry (Crocker *et al.*, 1988).

Often AgNORs are tightly clustered. The low counts scored in benign lesions probably represent AgNOR clusters, and the high counts for malignant cells result from a combination of multiple AgNOR clusters and dispersed AgNORs (Underwood & Giri, 1988; Crocker *et al.*, 1989). In tumour cells under different growth conditions, the number and morphology of NORs show variation. A close relationship between AgNOR clusters and AgNORs would seem likely. However, there are few reports on morphological changes of AgNORs and AgNOR clusters under different growth conditions. Also the term AgNOR has been frequently confused with 'AgNOR cluster' and some authors have apparently regarded one AgNOR cluster as one AgNOR. Indeed, it may not be easy to discern AgNORs from AgNOR clusters on conventional paraffin sections. It is important, however, to make this distinction to facilitate further understanding of NORs. To clarify the relationship between AgNORs, AgNOR clusters and cell proliferation, we investigated the changes in localisation and number of AgNORs and AgNOR clusters in HeLa cells during serum starvation.

### Materials and methods

HeLa S3 cells were grown both on chamber slides (Nunc, Inc. USA) and 100 mm tissue culture dishes for 48 h in Dulbecco's modified Eagle's medium (DMEM) supplemented with 10% calf serum in a humidifed atmosphere of 5% $CO_2$ in air. At the end of this time, the medium was replaced by DMEM without calf serum. The cells were incubated in serum-free medium for 24, 48 or 72 h. Control cells were incubated in medium containing the serum. The chamber slides were washed with PBS (pH 7.2) and dried well in cool air. The cells were fixed with 3:1 ethanol/acetic acid solution, and staining for AgNORs was performed as previously described (Ploton *et al.*, 1986). Briefly, a silver colloid solution was prepared by dissolving gelatin in 1% aqueous formic acid at a concentration of 2 per cent. This solution was mixed in a proportion of 1:2 by volume with 50% aqueous silver nitrate to obtain a final working solution. Silver staining was performed by immersing the slide in the solution at room temperature for 30 min. After staining, the specimens were washed in running deionised water for 10 min. Counter staining was not performed, and slides were dehydrated with ethanol and mounted in a synthetic medium.

Two methods of counting black dots within nuclei were employed as recently reported (Crocker *et al.*, 1989). One method involved counting AgNOR clusters (almost aggregated or partly disaggregated nucleoli) within nuclei and making no attempt to resolve the clustered subsidiary small black dots (AgNORs) that can be seen within AgNOR clusters. The number of AgNOR clusters was easily counted. The second method involved counting all of the small black dots, AgNORs, even though they were present in AgNOR clusters. In this method, therefore, an attempt was made actually to count individual AgNORs. Careful focusing of the microscope allowed the AgNORs to be visualised as individual black dots. Furthermore, we also added a third method. This involved counting individual 'satellite' AgNORs, which were different from the AgNOR dots seen within AgNOR clusters. The satellite AgNORs were dispersed as single dots within the cell nucleus. At least 200 nuclei were examined using a × 100 oil immersion objective to a total magnification of ×1000. The dots were counted using a simple eyepiece graticule to prevent recounting. Tumour cells were taken at random for the counting procedure. The mean number of AgNOR clusters, the mean number of satellite AgNORs and the mean total of individual AgNORs were calculated for each cell. The data were subjected to analysis by Student's *t*-test.

The cells in tissue culture dishes were harvested with a trypsin-EDTA solution and washed well in cold PBS. The cell number was determined using a Coulter counter (Model ZB, Coulter Electronics, Inc., USA). Flow cytometric DNA measurement was carried out with a FACS Analyser (Becton Dickinson, USA) for analysis of the cell cycle distribution of HeLa cells under the different culture conditions. The cell suspensions, which had been used for determining the growth curves, were centrifuged and resuspended in a Triton X-100 solution (0.1% in PBS, Sigma Chemical Co., USA), treated with 0.1% RNase (Sigma Chemical Co., USA), and stained with 50 $\mu g\ ml^{-1}$ of propidium iodide (PI, Calbiochem Co., USA) as described previously (Sasaki *et al.*, 1984). Nuclear DNA content was measured with the FACS Analyser, which was calibrated using fluorescence beads. The coefficient of variation (CV) was less than 2 per cent.

### Results

The distribution of AgNORs in HeLa cells following serum starvation is shown in Figure 1. The well-defined black silver dots were observed in all nuclei of HeLa cells grown on chamber slides for 48 h in DMEM supplemented with 10% calf serum (Figure 1a). On the other hand, the AgNORs tended to be clustered after 72 h of incubation in a serum-free medium. The AgNOR dots and AgNOR clusters were more aggregated to form larger groups of small dots in HeLa cells cultured in a serum-free medium than in those in serum-containing medium (Figure 1b). The cells grown in serum-deprived conditions were stained with hematoxylin, and no apparent microscopic changes were seen in the morphology of the nucleoli and nuclei.

The growth rate of cells in DMEM supplemented with calf serum was faster than that in serum-free medium with doubling times of 30 h and 48 h respectively (Figure 2). In the cells grown for 48 h in DMEM supplemented with calf serum, the percentage of cells in the S and G1/0 phases was estimated by FCM to be 35.8 and 49.5% (Figure 3a). In contrast, after 72 h of incubation in serum-free medium the proportion of cells in S-phase was reduced to 24.7%, and the proportion in

Correspondence: K. Matsumura.
Received 1 August 1989; and in revised form 26 February 1990.

G1/0 phase was increased to 68.0% (Figure 3b).

The number of AgNORs was not significantly altered during serum-free culture: the mean number of AgNORs per nucleus was 13.2 dots (Figure 4a). The number of satellite AgNORs was also not significantly changed during serum-free culture (Figure 4c). In contrast, the number of AgNOR clusters was significantly decreased under such conditions. The mean number of AgNOR clusters in HeLa cells cultured in the presence of serum and after 72 h of incubation in serum-free medium was 4.9 and 2.9, respectively (Figure 4b).

**Figure 1** Morphological changes of NORs in HeLa cells during serum starvation. **a**, NOR staining of HeLa cells grown for 48 h in DMEM supplemented with 10% calf serum. **b**, NOR staining of HeLa cells after 72 h of incubation in a serum-free medium. Original magnifications × 280; inset, × 700.

**Figure 2** Growth curves of HeLa cells. Cells grown in DMEM in the absence (●) or presence (■) of serum. Values are the mean of three separate experiments.

**Figure 3** Changes of DNA distribution analysed by flow cytometry in HeLa cells during serum starvation. **a**, DNA histogram of HeLa cells grown under the same conditions as in Figure 1a. **b**, DNA histogram of HeLa cells grown under the same conditions as in Figure 1b.

**Figure 4** Changes in the mean number of AgNORs **a**, AgNOR clusters **b**, and satellite AgNORs **c**, in HeLa cells in DMEM supplemented with calf serum (dashed lines) and following serum deprivation (solid line). Although the number of AgNORs or satellite AgNORs was not significantly altered, the number of AgNOR clusters was significantly decreased after 48 and 72 h in serum-free medium (* = $p < 0.01$). Values are the mean ± s.e. of three separate experiments.

### Discussion

We have demonstrated that the aggregation of both AgNORs and smaller AgNOR clusters within a larger AgNOR cluster become evident during retardation of cell proliferation. The reasons for this observation are uncertain. However, cell proliferation seems to bear a relationship to the morphological changes of AgNORs (Stahl, 1982; Wachtler *et al.*, 1986). Following dimethylsulfoxide (DMSO) treatment of human promyelocytic leukaemia (HL60) cells, the number of detectable AgNORs fell rapidly, reaching levels characteristic of normal bone marrow cells. Suppression of rDNA transcription occurs and AgNOR staining reflects regulation of rRNA synthesis (Reeves *et al.*, 1984). Quiescent lymphocytes contain 1 or 2 AgNOR sites in number but multiple AgNOR sites following blast transformation. Such transformation could be regarded as enabling more rapid and wide spread rDNA cistron transcription with increasing cell activity and consequent protein synthesis (Field *et al.*, 1984). On the other hand, the mean number of total AgNORs per cell in our study was not changed in HeLa cells when cell proliferation was retarded by serum deprivation. There are few papers which have distinguished between numbers of AgNORs and numbers of AgNOR clusters. The mean number of total AgNORs in malignant breast lesions significantly exceeded that in normal breast and in benign lesions (Smith & Crocker, 1988). This perhaps indicates that malignant transformation is needed for the variation in AgNORs counts. A change in cell proliferation would not itself affect the total number of AgNORs, although it may change the number and morphology of AgNOR clusters.

The present study indicates that measurement of the number of AgNOR clusters may be a useful method of determining the rate of cell proliferation. Therefore, it will be of great interest to determine the effect of anticancer drugs, or radiation therapy on the number of AgNOR clusters.

This work was supported in part by a grant No. 01571094 from the Ministry of Education, Science and Culture of Japan.

### References

CROCKER, J., MACARTNEY, J.C. & SMITH, P.J. (1988). Correlation between DNA flow cytometric and nucleolar organizer region data in non-Hodgkin's lymphomas. *J. Pathol.*, **154**, 151.

CROCKER, J., BOLDY, D.A.R. & EGAN, M.J. (1989). How should we count AgNORs? Proposal for a standardized approach. *J. Pathol.*, **158**, 185.

FIELD, D.H., FITZGERALD, P.H. & SIN, F.Y.T. (1984). Nucleolar silver-staining patterns related to cell cycle phase and cell generation of PHA-stimulated human lymphocytes. *Cytobios.*, **41**, 23.

HALL, P.A., CROCKER, J., WATTS, A. & STANSFELD, A.G. (1988). A comparison of nucleolar organizer region staining and Ki67 immunostaining in non-Hodgkin's lymphoma. *Histopatholoy*, **12**, 373.

HOWAT, A.J., GIRI, D.D., WRIGHT, A.L. & UNDERWOOD, J.C.E. (1988). Silver-stained nucleoli and nucleolar organizer region counts are of no prognostic value in thick cutaneous malignant melanoma. *J. Pathol.*, **156**, 227.

MATSUMURA, K., SASAKI, K., TSUJI, T. & SHINOZAKI, F. (1989). The nucleolar organizer region associated protein (AgNORs) in salivary gland tumors. *Int. J. Oral Maxillofac. Surg.*, **18**, 76.

PLOTON, D., MENAGER, M., JEANNESSON, P., HIMBER, G., PIGEON, F. & ADNET, J.J. (1986). Improvement in the staining and in the visualization of the argyrophilic proteins of the nucleolar organizer region at the optical level. *Histochem. J.*, **18**, 5.

REEVES, B.R., CASEY, S., HONEYCOMBE, J.R. & SMITH, S. (1984). Correlation of differentiation state and silver staining of nucleolar organizers in the promyelocytic cell line HL-60. *Cancer Genet. Cytogenet.*, **13**, 159.

SASAKI, K., OGINO, T., NAGAMINE, Y. & TAKAHASHI, M. (1984). Fundamental study for flow cytometric DNA analysis using propidium iodide. *J. Jpn. Clin. Cytol.*, **23**, 1.

SMITH, R. & CROCKER, J. (1988). Evaluation of nucleolar organizer region-associated protein in breast malignancy. *Histopathology*, **12**, 113.

STAHL, A. (1982). The nucleolus and nucleolar chromosomes. In: *The Nucleolus*, Jordan, E.G. & Cullis, C.A. (eds) p. 1. Cambridge University Press: Cambridge.

SUAREZ, V., NEWMAN, J., HILEY, C., CROCKER, J. & COLLINS, M. (1989). The value of NOR numbers in neoplastic and non-neoplastic epithelium of the stomach. *Histopathology*, **14**, 61.

UNDERWOOD, J.C.E. & GIRI, D.D. (1988). Nucleolar organizer regions as diagnostic discriminations for malignancy. *J. Pathol.*, **155**, 95.

WACHTLER, F., HOPMAN, A.H.N., WRIGHT, J. & SCHWARZACHER, H.G. (1986). On the position of nucleolus organizer regions (NORs) in interphase nuclei. *Exp. Cell Res.*, **167**, 227.


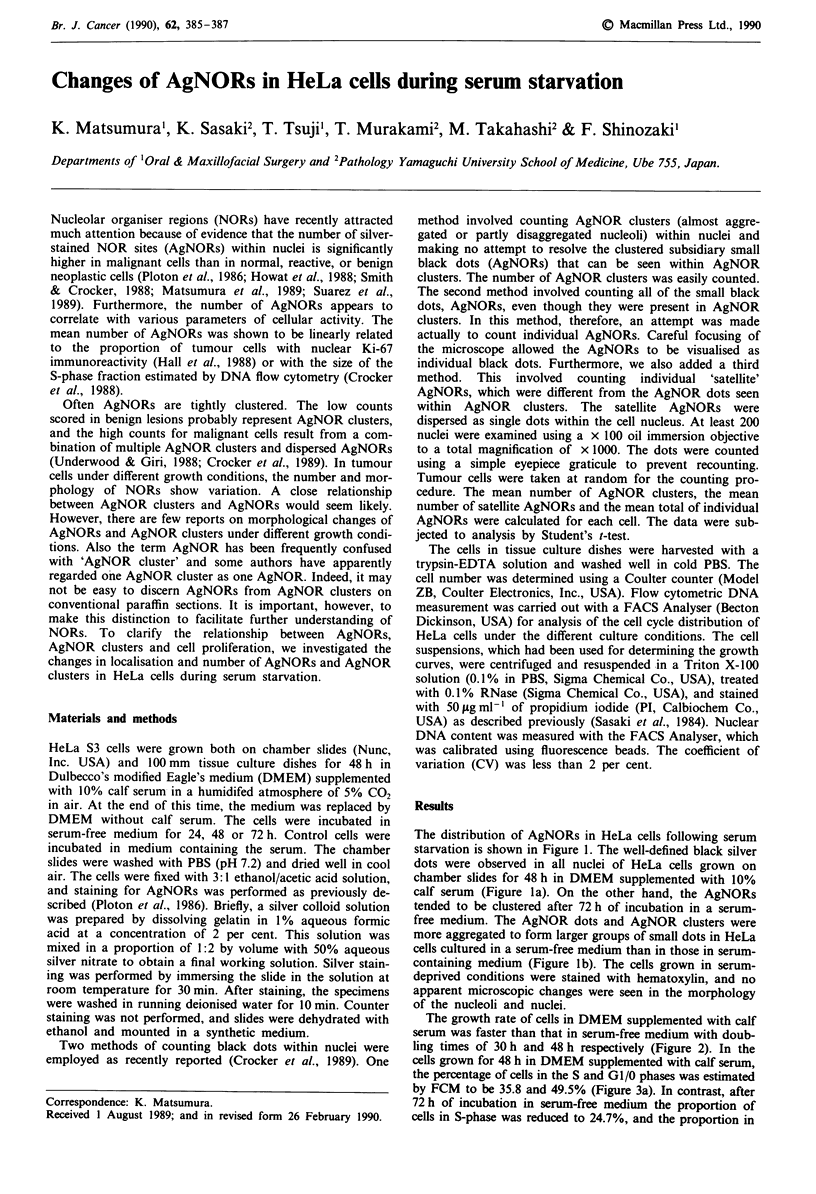

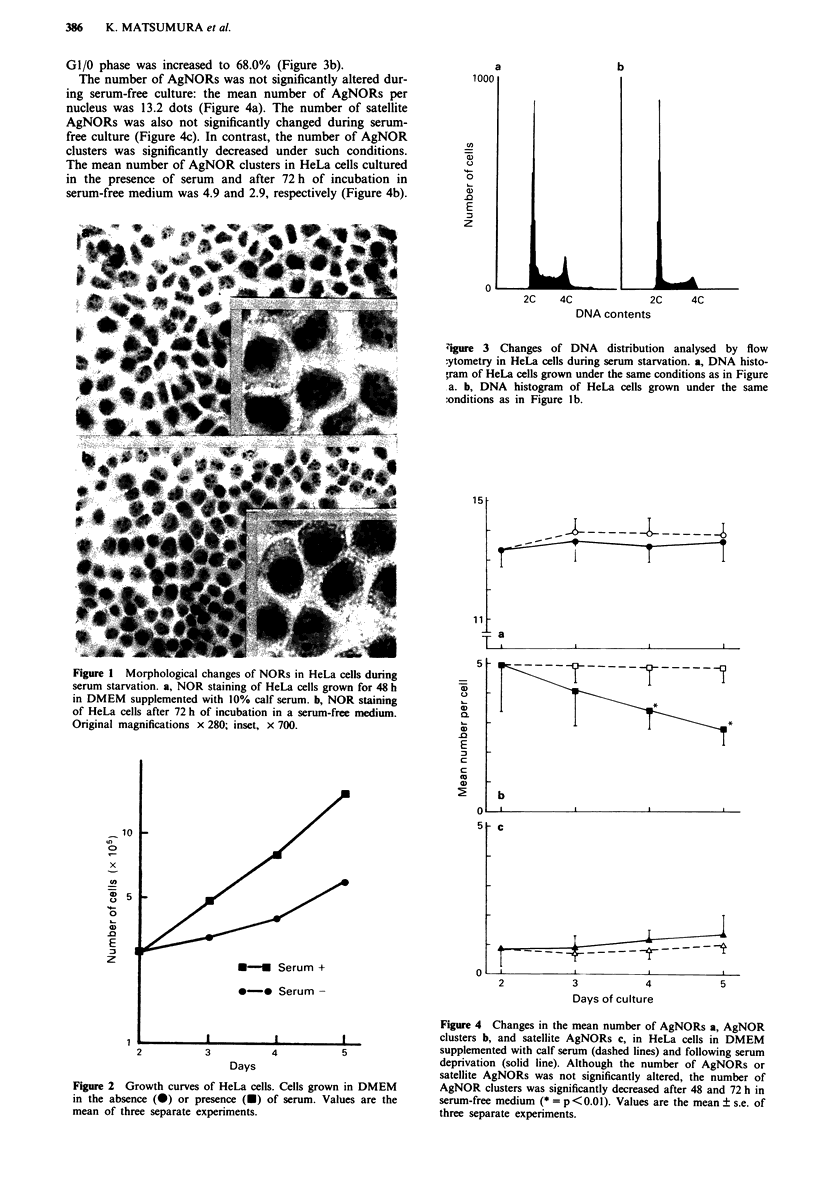

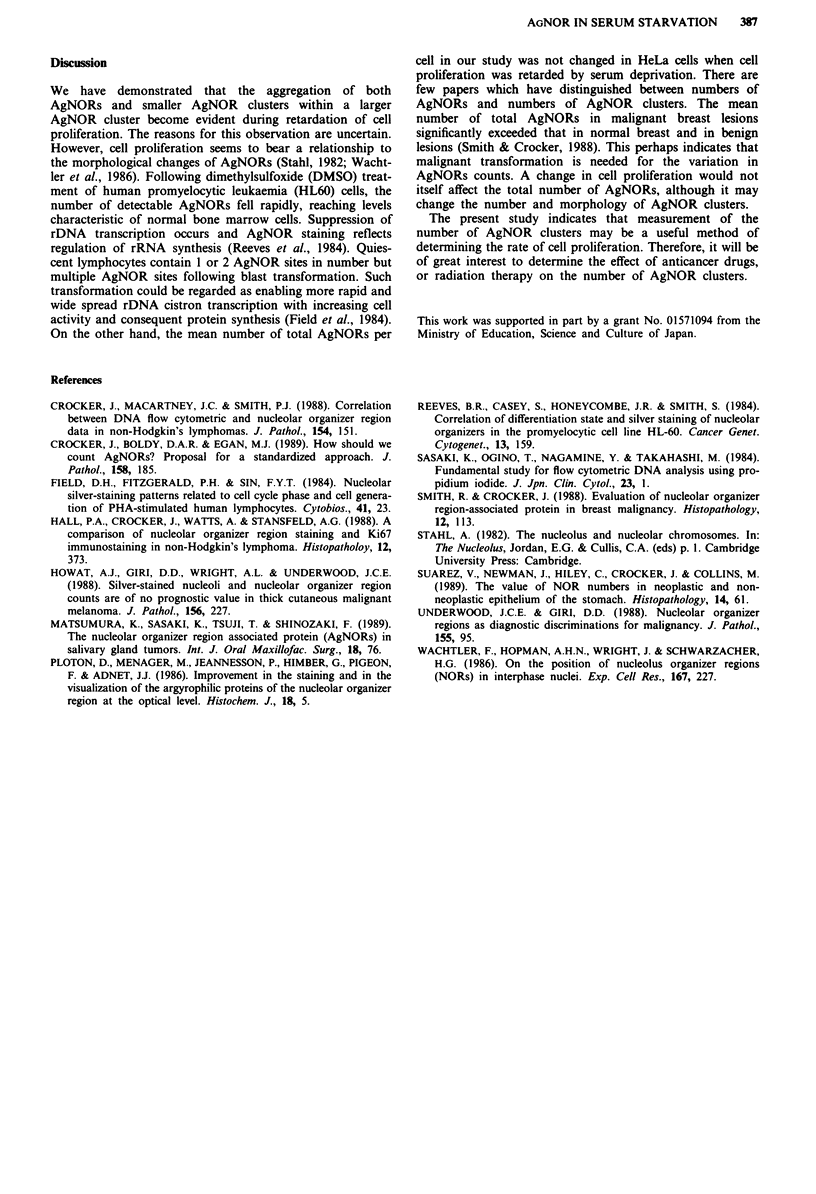


## References

[OCR_00329] Crocker J., Boldy D. A., Egan M. J. (1989). How should we count AgNORS? Proposals for a standardized approach.. J Pathol.

[OCR_00324] Crocker J., Macartney J. C., Smith P. J. (1988). Correlation between DNA flow cytometric and nucleolar organizer region data in non-Hodgkin's lymphomas.. J Pathol.

[OCR_00334] Field D. H., Fitzgerald P. H., Sin F. Y. (1984). Nucleolar silver-staining patterns related to cell cycle phase and cell generation of PHA-stimulated human lymphocytes.. Cytobios.

[OCR_00338] Hall P. A., Crocker J., Watts A., Stansfeld A. G. (1988). A comparison of nucleolar organizer region staining and Ki-67 immunostaining in non-Hodgkin's lymphoma.. Histopathology.

[OCR_00344] Howat A. J., Giri D. D., Wright A. L., Underwood J. C. (1988). Silver-stained nucleoli and nucleolar organizer region counts are of no prognostic value in thick cutaneous malignant melanoma.. J Pathol.

[OCR_00350] Matsumura K., Sasaki K., Tsuji T., Shinozaki F. (1989). The nucleolar organizer regions associated protein (Ag-NORs) in salivary gland tumors.. Int J Oral Maxillofac Surg.

[OCR_00355] Ploton D., Menager M., Jeannesson P., Himber G., Pigeon F., Adnet J. J. (1986). Improvement in the staining and in the visualization of the argyrophilic proteins of the nucleolar organizer region at the optical level.. Histochem J.

[OCR_00361] Reeves B. R., Casey G., Honeycombe J. R., Smith S. (1984). Correlation of differentiation state and silver staining of nucleolar organizers in the promyelocytic leukemia cell line HL-60.. Cancer Genet Cytogenet.

[OCR_00372] Smith R., Crocker J. (1988). Evaluation of nucleolar organizer region-associated proteins in breast malignancy.. Histopathology.

[OCR_00382] Suarez V., Newman J., Hiley C., Crocker J., Collins M. (1989). The value of NOR numbers in neoplastic and non-neoplastic epithelium of the stomach.. Histopathology.

[OCR_00387] Underwood J. C., Giri D. D. (1988). Nucleolar organizer regions as diagnostic discriminants for malignancy.. J Pathol.

[OCR_00392] Wachtler F., Hopman A. H., Wiegant J., Schwarzacher H. G. (1986). On the position of nucleolus organizer regions (NORs) in interphase nuclei. Studies with a new, non-autoradiographic in situ hybridization method.. Exp Cell Res.

